# Effect of tongue hygiene instruction on periodontal patients: an experimental study

**DOI:** 10.1186/s12903-025-07618-3

**Published:** 2026-01-09

**Authors:** Fazele Atarbashi-Moghadam, Sajjad Salari, Seyed Sepehr Mirebeigi-Jamasbi

**Affiliations:** 1 Department of Periodontology, School of Dentistry, Shahid Beheshti University of Medical Sciences, Tehran, Iran; 2 School of Dentistry, Shahid Beheshti University of Medical Sciences, Daneshju Blvd., Velenjak St., Chamran Highway, Tehran, 1983963113 Iran

**Keywords:** Tongue, Oral hygiene, Prevalence, Periodontitis, Gingivitis

## Abstract

**Background:**

Although tongue cleaning provides various benefits such as reducing dental plaque, improving halitosis, managing gingival inflammation, and enhancing subjective taste, many people do not include tongue hygiene in their oral health habits. This study aimed to assess the frequency of tongue cleaning and the effect of tongue hygiene instruction on oral hygiene habits among periodontal patients.

**Methods:**

A total of 222 patients, diagnosed with gingivitis or periodontitis, participated in this study. Participants completed a questionnaire before and one month after receiving tongue hygiene instruction. The questionnaire included demographic information and a section for tongue cleaning habits. The instruction was delivered using educational models and a leaflet prepared by the authors. Data were analyzed using chi-square, Fisher’s exact test, independent samples t-test, Wilcoxon signed-rank test, and McNemar test, with significance set at *P* < 0.05.

**Results:**

Initially, 32.9% of participants practiced tongue cleaning, with women (68.5%, *P* < 0.001), non-smokers (91.8%, *P* < 0.001), and patients with gingivitis (69.9%, *P* < 0.001) showing significantly higher rates. After instruction, the prevalence of tongue cleaning increased to 76.2%, with daily cleaning rising from 11.2% to 45.4% (*P* < 0.001). The instruction was particularly effective for participants with periodontitis (increased from 35.4% to 51.4%) and those without a college education (52.1% to 70.6%), with both being statistically significant (*P* < 0.001). Additionally, 42.6% of participants reported improved taste perception, particularly among those over 60 years of age (*P* < 0.001).

**Conclusion:**

Tongue hygiene instruction significantly improved tongue cleaning habits and oral hygiene among periodontal patients, emphasizing the importance of targeted educational interventions. Tongue hygiene instructions should be a part of routine dental care to raise patient awareness, which in turn promotes better adherence to the practice.

**Supplementary Information:**

The online version contains supplementary material available at 10.1186/s12903-025-07618-3.

## Background

The tongue, a crucial organ within the oral cavity, plays a significant role in various functions such as speech, swallowing, taste perception, and jaw development [1]. Its dorsal surface, comprising nearly one-third of the oral cavity’s surface, is characterized by a papillary structure which predisposes it to the accumulation of small particles [2]. The dorsal surface of the tongue typically appears pink and may have a thin white layer [1]. A common condition affecting this surface is tongue coating, a grayish-white deposit comprising oral bacteria, desquamated epithelial cells, and food debris [3]. Although the tongue is susceptible to various physiological and pathological conditions, such as vascular or reactive lesions and both benign and malignant tumors, tongue coating remains the most prevalent condition, with its frequency differing across regions [1, 4, 5].

The papillary surface of the tongue provides an ideal environment for microbial accumulation and biofilm formation [6]. It harbors the highest bacterial load of all oral tissues, accommodating nearly two-thirds of the total oral microbiota. The dominant species include aerobic and facultative anaerobic streptococci, such as Streptococcus salivarius, S. mitis, and S. sanguis, while Veillonella species often inhabit the crypts of the papillae [7]. Such microbial communities can act as reservoirs for cariogenic and periodontopathogenic bacteria, including S. mutans, P. gingivalis, and Aggregatibacter actinomycetemcomitans, linking the tongue coating not only to local oral diseases such as dental caries, periodontitis, peri-implantitis, and halitosis, but also to potential systemic inflammatory effects through chronic cytokine activity [6–8]. The coated tongue is therefore recognized as the primary source of oral malodor and a reservoir for periodontal pathogens [9, 10]. Furthermore, halitosis can lead to embarrassment, social withdrawal, occasional communication difficulties, and hinder personal relationships [11].

Oral hygiene exerts a significant influence on the formation of tongue coating [3]. Incorporating mechanical tongue cleaning into oral hygiene practices is essential, particularly for patients with oral malodor and periodontal diseases [9]. Several cleaning tools are available on the market, including tongue brushes, scrapers, toothbrushes, mouthwashes, and specialized pastes that help dislodge the adhering biofilm [8, 10]. However, studies have yielded contrasting results, and there is still no consensus on the most effective tool and technique for tongue cleaning [11, 12]. Nevertheless, toothbrushes are the most commonly used tools for tongue cleaning [13].

Besides tongue cleaning, the use of probiotics, postbiotics, and paraprobiotics has been proposed as an adjunctive approach to modulate the oral microbiota and enhance halitosis control. These agents may exert antimicrobial, anti-inflammatory, and immunomodulatory effects while neutralizing acidic pH and reducing oxidative stress, thus supporting oral health and odor prevention [14].

Tongue cleaning offers several benefits, such as reducing dental plaque, improving halitosis, managing gingival inflammation, enhancing subjective taste, and promoting better digestive health [6, 15, 16]. Despite these advantages, many people still neglect tongue hygiene. The reasons may be a lack of awareness, difficulties in accomplishing the task, and the inability to afford the cleaning devices [17].

Previous studies have reported varying rates of tongue-cleaning practices across different populations [2, 13, 16]. However, to our knowledge, no research has yet examined the impact of tongue hygiene instruction on these habits. Therefore, this study aimed to determine the frequency of tongue cleaning and assess the effect of tongue cleaning instruction on the oral hygiene habits of periodontal patients referred to the Department of Periodontics at Shahid Beheshti University of Medical Sciences. The null hypothesis stated that tongue hygiene instruction would not significantly affect the oral hygiene behavior of periodontitis patients.

## Methods

This study was designed and conducted following the Declaration of Helsinki [18]. It was approved by the Ethics Committee of Shahid Beheshti University of Medical Sciences (IR.SBMU.DCR.REC.1401.031). A Persian-language questionnaire was developed, including demographic information (age, gender, education, smoking status, and history of systemic diseases), as well as eight questions specifically related to tongue hygiene. Written informed consent was obtained from the patients for the use of their answers for research purposes without revealing their identity.

To validity of the questionnaire was assessed using the content validity index (CVI) and the content validity ratio (CVR). The CVI was evaluated based on the relevance, clarity, and simplicity of the questions, while the CVR was calculated using the Lawshe equation. For the questionnaire to be considered valid, thresholds of CVI > 0.79 and CVR > 0.62 were required. To determine reliability, fifty individuals completed the questionnaire twice, with a 10-day interval between the two administrations. Reliability was confirmed with a kappa statistic, greater than 0.80.

Study participants were patients referred to the Department of Periodontics at Shahid Beheshti University of Medical Sciences, who were over 18 years of age and diagnosed with gingivitis or periodontitis based on the 2017 World Workshop [19]. A patient was considered a periodontitis case if interdental clinical attachment loss (CAL) was detectable at ≥ 2 non-adjacent teeth, or if buccal/oral CAL ≥ 3 mm with pocketing > 3 mm was present at ≥ 2 teeth, and the CAL could not be attributed to non-periodontal causes (e.g., traumatic recession, cervical caries, distal second molar defects caused by third molar malposition/extraction, endodontic lesions, or vertical root fractures). Additionally, patients who did not meet the criteria for periodontitis and presented with bleeding on probing (BOP) were classified as having gingivitis [19]. After explaining the study objectives and obtaining written informed consent, eligible participants were enrolled. Patients with mental or physical disabilities that could impair their ability to follow the hygiene instructions were excluded from the study.

All participants completed the initial questionnaire after receiving non-surgical periodontal therapy including scaling, root planning, and standard oral hygiene instructions. Specific education on tongue cleaning was then provided. Patients were informed about its importance and benefits, the available tools (toothbrushes and tongue scrapers), and the proper tongue cleaning method. These instructions were delivered through educational models and a leaflet developed by the authors, based on evidence from previous studies [20–23]. Participants were encouraged to retain the leaflet for future reference. One month later, participants were contacted to complete a follow-up survey including three of the initial questions and an additional yes/no question about experiencing any taste improvement.

The required sample size was calculated to be 222 participants to achieve a 95% confidence level in estimating the prevalence of tongue cleaning. This calculation was based on a margin of error of 0.06 and a presumed prevalence of 0.3 [13]. Data were analyzed using SPSS 26 software, with a significance level set at *P* < 0.05. Associations between having a tongue cleaning habit or awareness of its importance and the study variables were analyzed using Chi-square test, Fisher’s exact test, and independent samples t-test as appropriate. The effect of the instruction on habit formation and cleaning frequency was assessed using McNemar test and Wilcoxon signed-rank test, respectively. In addition, logistic regression analysis was conducted to examine whether the change in tongue cleaning habit was influenced by the study variables.

## Results

The CVR value for the questionnaire was 0.9, and the CVI values were 0.97 for relevance, 0.94 for clarity, and 0.97 for simplicity. All these values surpassed the required thresholds. Furthermore, the reliability of the questionnaire was confirmed with a kappa statistic of 0.959.

Among the 222 patients who completed the questionnaire, 107 (48.2%) were males and 115 (51.8%) were females, with mean ages of 42.3 ± 14.7 and 41.9 ± 15.0 years, respectively. Table 1 summarizes the demographic data and periodontal conditions of the participants.

**Table 1:** Summary of the participants’ demographic data and periodontal conditions

Patients	Number (%)	Sex		Mean age (SD)	Smoking history		Education level		Periodontal condition	
		Male (%)	Female (%)		Positive (%)	Negative (%)	University (%)	Lower (%)	Gingivitis (%)	Periodontitis (%)
With tongue cleaning	73 (32.9)	23 (31.5)	50 (68.5)	41.7 (15.4)	6 (8.2)	67 (91.8)	39 (53.4)	34 (46.6)	51 (69.9)	22 (30.1)
Without tongue cleaning	149 (67.1)	84 (56.4)	65 (43.6)	42.3 (14.6)	40 (26.8)	109 (73.2)	19 (12.8)	130 (87.2)	44 (29.5)	105 (70.5)
Total	222 (100)	107 (48.2)	115 (51.8)	42.1 (14.9)	46 (20.7)	176 (79.3)	58 (26.1)	164 (73.9)	95 (42.8)	127 (57.2)

*SD* Standard deviation. Data are presented as numbers and percentages

**Fig. 1:**
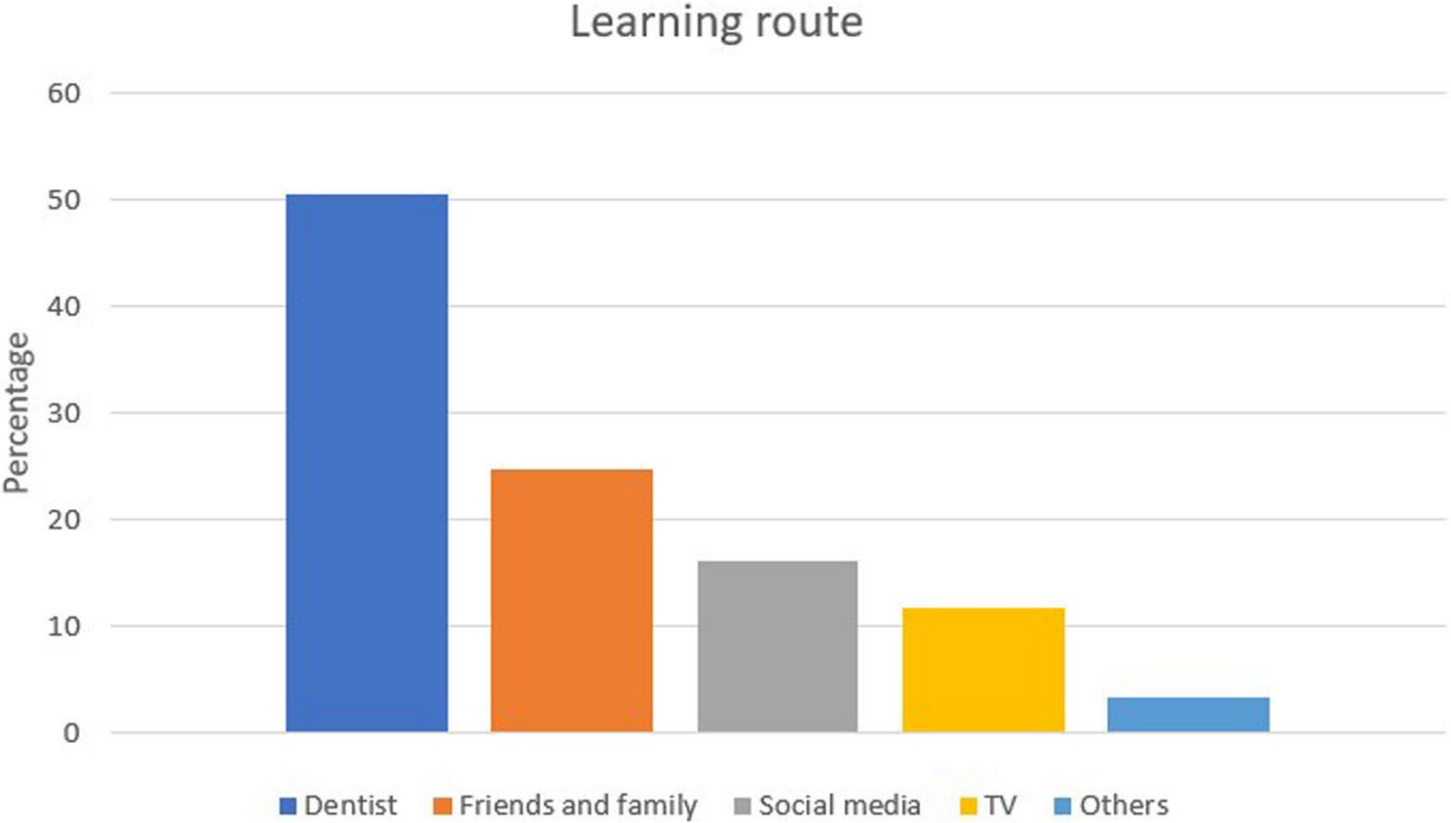
Sources of tongue hygiene instruction among participants. This figure illustrates the distribution of participants according to where they had previously received information about tongue cleaning. Percentages represent the proportion of respondents reporting each source

Overall, 41.9% of the participants were aware of the importance of tongue cleaning. This awareness was closely linked to their education level (p-value < 0.001). Among those familiar with the necessity and benefits of tongue cleaning, the most recognized benefit was the reduction of breath malodor (68.6%), followed by pigment removal from the tongue surface (60.4%). 91.4% of respondents stated that the toothbrush was the tool they knew for tongue cleaning. Participants had received information about tongue cleaning mostly from dentists (50.6%), followed by friends and family (25%) (Fig. 1).

Before receiving tongue cleaning instruction, 73 patients (32.9%) reported cleaning their tongues, with varying frequencies of daily (31.5%), occasionally (50.7%), and rarely (17.8%). Among them, 94.5% used toothbrushes for this purpose, while others used tongue scrapers. Tongue cleaning was significantly more prevalent among women (68.5%, *P* < 0.001), non-smokers (91.8%, *P* < 0.001), and in patients with gingivitis (69.9%, *P* < 0.001). Specifically, 82.6% of patients with periodontitis did not practice tongue cleaning, compared to 46.3% of those with gingivitis. Education level also showed a significant difference (*P* < 0.001), with 67.2% of those with a college education or higher practicing tongue cleaning, compared to only 20.7% of those with lower education levels. However, there was no significant difference in tongue cleaning habits between individuals older and younger than 60 years (*P* = 0.55).

One month after the tongue-cleaning instruction, only 143 patients completed the follow-up questionnaire. To ensure accurate evaluation, only these 143 patients who completed both the pre-and post-instruction questionnaires were included in the analysis of the instruction’s effects (Table 2). Among these participants, 76.2% reported following tongue-cleaning habits after instruction, while 23.8% did not adopt the habit even after instruction. Notably, 64.2% of those who initially did not clean their tongue began doing so (*P* < 0.001). The frequency of tongue cleaning also increased significantly, with daily cleaning rising from 11.2% to 45.4% (*P* < 0.001). Tongue-cleaning habits significantly increased among participants with periodontitis from 35.4% to 51.4%, and among non-college education participants from 52.1% to 70.6% (*P* < 0.001 for both). However, logistic regression showed that the influence of tongue-cleaning instruction was not dependent on age, gender, education, and smoking (*P* > 0.05). Among the participants who started cleaning their tongues after the instruction, 42.6% reported an improvement in their sense of taste. This change was significantly relared to age, as all individuals over 60 years old experienced enhanced taste perception (*P* < 0.001).

**Table 2:** Frequency of tongue cleaning among participants before and after instruction

Timepoint	Tongue cleaning frequency	number	percentage
Before instruction	Everyday	16	11.2
	Occasionally	24	16.8
	Rarely	8	5.6
	Never	95	66.4
After instruction	Everyday	65	45.5
	Occasionally	41	28.7
	Rarely	3	2.1
	Never	34	23.8
Total		143	100

* This table compares the frequency of tongue cleaning reported by the 143 participants who completed both baseline and follow-up questionnaires. Post-instruction data reflect the responses collected one month after the educational intervention

## Discussion

This study investigated the prevalence of tongue cleaning among periodontal patients and the impact of tongue hygiene instruction on this habit. Among the 222 participants, 32.9% reported cleaning their tongues, with 31.5% doing so daily. These findings are consistent with previous studies reporting comparable or slightly higher rates in different populations. For instance, in a Japanese group of healthy individuals, 37% engaged in tongue cleaning, with 24.6% practicing it daily [13]. In an Italian population, 39% had a tongue-cleaning habit, with 64% performing it daily [24]. In a hospital setting in Japan, 52.6% of the patients practiced tongue cleaning, with 35.1% doing so daily [16]. In a Nepalese dental school population, 53.8% reported tongue cleaning, with 69% engaging daily [2]. These findings indicate that, despite its benefits, tongue cleaning remains neglected across various countries and communities.

The prevalence of tongue cleaning in the present study was higher among women than men, aligning with Kishi et al. [13] and Matsuda et al. [16]; while Yadav et al. [2] reported a nearly equal ratio between genders. It has been shown that oral hygiene practices differ between men and women, with women generally demonstrating better oral hygiene, more consistent oral care habits, and more regular dental visits [25]. Conversely, men are more likely to neglect oral health, leading to a higher incidence of periodontal disease, oral cancer, and dental trauma [26]. Therefore, the higher prevalence of tongue cleaning among women in our study likely reflects their greater attention to oral hygiene.

Tongue cleaning was significantly more common among individuals with gingivitis compared to those with periodontitis, and among patients with a university education compared to those with non-college education. In this study, 58% of participants lacked awareness of the benefits of tongue cleaning, which likely contributed to the low prevalence of this practice. Dentists were the primary source of information about tongue cleaning, highlighting their critical role in oral health education. Similarly, Mueller et al. demonstrated that people received basic oral health education mostly from dentists, followed by oral hygienists in schools or kindergartens [27].

Consistent with other studies [2, 13, 24], the toothbrush was the most commonly used tool for tongue cleaning, while fewer than 10% of participants were aware of other tools. Despite the effectiveness of specialized tongue-cleaning devices, fewer people are familiar with them. While toothbrushes are designed for cleaning hard, immobile surfaces, tongue cleaners are specifically designed to clean the soft and flexible surface of the tongue, making them more effective at tongue coating removal [28].

In this study, tongue-cleaning instruction led to a significant increase in its practice among participants who completed both questionnaires, rising from 33.6% at the start of the study to 76.2% one month after the instruction. Notably, 64.2% of those who initially did not clean their tongues adopted the habit following the instruction. This improvement might be attributed to several mechanisms. First, the educational intervention raised patients’ awareness of the importance of tongue hygiene, as less than half of the participants were initially familiar with its significance. Second, the instruction introduced patients to various tools for tongue cleaning, as over 90% of the participants were initially unaware of tongue scrapers. Furthermore, by demonstrating the proper tongue-cleaning technique, the instruction may have helped patients reduce the gag reflex and discomfort commonly associated with this practice. Therefore, both increased awareness and practical facilitation contributed to the behavioral improvement observed after the intervention.

Among the participants who started cleaning their tongues after receiving instruction, 42.6% reported an enhanced taste perception. This improvement was consistent across sex and education levels but varied with smoking status and age. Notably, 88.5% of those who reported better taste perception were non-smokers. Age also played a role, with all participants over 60 years, 54.5% of those aged 41–60, and only 19.4% of those under 41 years experiencing improved taste. These findings align with Madiloggovit et al. [29], who observed continuous tongue brushing over three months improved taste perception in 74% of older adults. Seerangaiyan et al. also demonstrated that tongue cleaning enhances saltiness perception, potentially reducing salt intake [30]. It is important to note that in the current study, the improvement in taste was based solely on self-reported perception and was not clinically evaluated; therefore, this finding should be interpreted with caution and warrants further clinical investigation to draw more definitive conclusions. A study suggested that excessive tongue cleaning could reduce the number of fungiform papillae and taste buds, negatively affecting taste sensation [31]. Currently, there is insufficient evidence to recommend an optimal frequency, duration, or method for tongue cleaning [21].

In this study, the frequency of tongue cleaning also increased significantly after receiving instruction (*p* < 0.001), with daily cleaning rising from 11.2% to 45.5% after the instruction. Among participants who previously did not clean their tongues, 56.5% of periodontitis patients and 60.5% of individuals without a college education, adopted the habit after the instruction. Although we did not find a comparable study that specifically measured the impact of tongue cleaning instruction on the prevalence of this practice, our findings align with behavioral health models that demonstrate the importance of awareness and health guidance in promoting behavior changes [32, 33].

A limitation of this study is the inability to accurately assess the specific impact of tongue cleaning on bad breath. As the participants received both scaling and root planning treatment and oral and tongue hygiene instruction at the same time, it was challenging to distinguish which intervention affected the reduction of bad breath. Another limitation is the use of self-reported data, which may be subject to reporting bias. Additionally, there was a notable loss to follow-up, as 64% of enrolled patients completed the post-intervention questionnaire. This could introduce selection bias, since the patients who completed the study may differ in motivation or compliance compared to those who did not.

Furthermore, this study did not include a separate control group receiving only general oral hygiene instructions without specific tongue-cleaning education. However, since conventional oral hygiene instructions generally do not include tongue-cleaning recommendations, minimal behavioral change would be expected in such a group. The current design allowed us to maximize the number of participants receiving special tongue-cleaning education and better observe the behavioral change effect.

Future studies with larger samples could incorporate both control and intervention groups to more precisely determine the causal impact of tongue-cleaning instruction. Additionally, studies with clinical examinations, microbiological analyses, and lower dropout rates could provide more comprehensive and reliable results. Furthermore, studies could evaluate the clinical impact of tongue hygiene on gingival and periodontal health, as well as explore the combined effects of tongue cleaning and the use of natural antimicrobial or probiotic substances to promote a more balanced oral microbiota.

## Conclusion

In conclusion, this study suggests that tongue cleaning education significantly improves oral hygiene practices among patients with periodontal diseases. The findings highlight the critical role of dentists and dental hygienists in oral health education and suggest that raising awareness about the importance of tongue cleaning could further enhance hygiene practices. The significant increase in tongue cleaning habits following instruction, particularly among those without a university degree, underscores the importance of targeted educational interventions in driving positive health behavior changes.

## Supplementary Information

Supplementary Material 1.

## Data Availability

The datasets used during the current study are available from the corresponding author on reasonable request.

## Abbreviations

CVI: Content validity index
CVR: Content validity ratio
