# Supplementary material for: Effect of tongue hygiene instruction on periodontal patients: an experimental study
Source: BMC Oral Health. 2026 Jan 9;26:295. doi: 10.1186/s12903-025-07618-3 (PMC12903663; doi:10.1186/s12903-025-07618-3)
Supplement: Supplementary file 1 — Supplementary Material 1. [file 12903_2025_7618_MOESM1_ESM.docx]

This document contains both the Persian and English versions of the questionnaire used in the study titled "Effect of tongue hygiene instruction on periodontal patients." The questionnaire was designed to assess participants' demographic information and tongue cleaning habits before and after receiving hygiene instructions. The content validity and reliability of the questionnaire were evaluated and confirmed through standardized methods, ensuring its accuracy for data collection. Below, the English version is presented first, followed by the Persian version.

**The English format of the questionnaires**

Part #1

Participant code: Gender: Male ⭘ Female ⭘ Age:……years
Education Level:……………… History of Systemic Diseases:…………..
Smoking: Yes⭘ No⭘ Periodontal Disease:………………

1. Have you ever heard about the importance of tongue-cleaning ?
   Yes □ No □
2. In your opinion, in which of the following situations is tongue cleaning helpful? (You can choose more than one option)
   a) Reducing bad breath □ b) Improving gum health □ c) Removing tongue stains□ d) Improving taste □ e) Improving digestive issues □
3. What tools do you know for tongue-cleaning?
4. How did you learn about tongue-cleaning?
   a) From my dentist □ b) from friends and family □
   c) On TV □ d) On social media □
   e) Other sources: ___________
5. Do you clean your tongue?
   Yes □ No □

If yes, please continue:

1. What tool do you use to clean your tongue?
2. How often do you clean your tongue?
   a) Every day □ b) occasionally □ c) Rarely □
3. What is your reason for cleaning your tongue?

Part #2

Participant code:

1. Do you clean your tongue?

Yes □ No □

If yes, please continue:

1. What tool do you use to clean your tongue?
2. How often do you clean your tongue?
   a) Every day □
   b) occasionally □
   c) Rarely □
3. Have you noticed any change in your sense of taste?
   Yes □ No □

If yes:

• Was the change overall or specific to a particular taste?

• On a scale of 1 (least) to 10 (most), how would you rate this change?

The Persian version:

# قسمت اول

شماره شرکت کننده جنسیت: مرد ⭘ زن ⭘ سن:..........سال

تحصیلات: ................. تاریخچه بیماری سیستمیک:

مصرف سیگار: بله ⭘ خیر⭘ نوع بیماری پریودنتال: ...................

1- آیا تاکنون در مورد ضرورت تمیز کردن زبان شنیده اید؟ بله □ خیر□

2- بنظر شما تمیز کردن زبان در کدامیک از شرایط زیر کمک کننده است؟ (می توانید بیشتر از یک مورد را انتخاب کنید)

الف) کاهش بوی بد دهان□ ب) بهبود بیماری های لثه □ ج) حذف رنگیزه از سطح زبان □

د) بهبود چشایی□ ه) بهبود مشکلات گوارشی□

3- چه وسایلی را برای تمیز کردن زبان می شناسید؟

4- اطلاعات مربوط به تمیز کردن زبان را از چه راهی بدست آورده اید؟

الف) دندانپزشک□ ب) دوستان و آشنایان□ ج) تلویزیون□ د) فضای مجازی□ ه) سایر موارد......

5- آیا زبان خود را تمیز می کنید؟ بله □ خیر □

در صورت مثبت بودن پاسخ،

6- با چه وسیله ای زبان خود را تمیز می کنید؟

7- چند وقت یکبار زبان خود را تمیز می کنید؟ هر روز □ گاهی□ بندرت□

8- دلیل شما برای تمیز کردن زبان چیست؟

# قسمت دوم

شماره شرکت کننده:

-1آیا بعد از آموزش بهداشت زبان، زبان خود را تمیز کرده اید؟

در صورت مثبت بودن پاسخ سوال قبل،

2- با چه وسیله ای زبان خود را تمیز می کنید؟

3- چند وقت یکبار زبان خود را تمیز می کنید ؟ هر روز □ گاهی□ بندرت□

4- آیا تغییری در وضعیت حس چشایی خود احساس کرده اید؟

در صورت مثبت بودن پاسخ :

- این تغییر به صورت کلی بوده یا حس چشایی خاصی تغییر کرده؟
- اگر بخواهید به این تغییر نمره دهید، از 1 تا 10 آن را چگونه ارزیابی می کنید؟
